# RBMX involves in telomere stability maintenance by regulating TERRA expression

**DOI:** 10.1371/journal.pgen.1010937

**Published:** 2023-09-27

**Authors:** Jingfan Liu, Tian Zheng, Dandan Chen, Junjiu Huang, Yong Zhao, Wenbin Ma, Haiying Liu

**Affiliations:** 1 MOE Key Laboratory of Gene Function and Regulation, School of Life Sciences, Sun Yat-sen University, Guangzhou, People’s Republic of China; 2 NHC Key Laboratory of Systems Biology of Pathogens, Institute of Pathogen Biology, Chinese Academy of Medical Sciences and Peking Union Medical College, Beijing, People’s Republic of China; Chinese Academy of Sciences, CHINA

## Abstract

Telomeric repeat-containing RNA (TERRA) is a class of long noncoding RNAs (lncRNAs) that are transcribed from subtelomeric to telomeric region of chromosome ends. TERRA is prone to form R-loop structures at telomeres by invading into telomeric DNA. Excessive telomere R-loops result in telomere instability, so the TERRA level needs to be delicately modulated. However, the molecular mechanisms and factors controlling TERRA level are still largely unknown. In this study, we report that the RNA binding protein RBMX is a novel regulator of TERRA level and telomere integrity. The expression level of TERRA is significantly elevated in RBMX depleted cells, leading to enhanced telomere R-loop formation, replication stress, and telomere instability. We also found that RBMX binds to TERRA and the nuclear exosome targeting protein ZCCHC8 simultaneously, and that TERRA degradation slows down upon RBMX depletion, implying that RBMX promotes TERRA degradation by regulating its transportation to the nuclear exosome, which decays nuclear RNAs. Altogether, these findings uncover a new role of RBMX in TERRA expression regulation and telomere integrity maintenance, and raising RBMX as a potential target of cancer therapy.

## Author summary

Telomeres are repetitive DNA: protein structures that protect linear chromosome ends from being recognized as double-strand DNA breaks. Telomeres bear very high loads of replication stress, and are essential for chromosomal stability and genome stability. Telomeric repeat-containing RNA (TERRA) is transcribed from subtelomeres to telomeres, and is prone to form R-loop structures by invading into telomeric DNA. In telomerase-negative human cancer cell lines, which maintain telomere length by homologous recombination-based alternative lengthening of telomeres (ALT) mechanism, both TERRA levels and telomeric R-loops are upregulated. Excessive telomere R-loops result in telomere instability. Therefore, only limited number of TERRA is required for telomere homeostasis. Here we show that, the RNA binding motif protein encoded on the X chromosome (RBMX), also known as hnRNP G, is a novel regulator of TERRA level and telomere integrity. Our data point to a completely new role of RBMX in TERRA expression regulation and telomere integrity maintenance, and open up a potential way for cancer therapy. In addition, this study also raises the possibility that RBMX also targeted other nuclear RNA to nuclear exosome for degradation.

## Introduction

In vertebrate species, linear chromosome ends are capped by a specific DNA/protein structure, which termed telomere. Telomeres are composed of TTAGGG/CCCTAA tandem DNA repeats and associated with telomere binding protein complex called shelterin [1,2]. Telomeres can protect eukaryotic chromosome ends from unnecessary degradation, attrition, promiscuous recombinogenic events, and prevent the chromosome ends from being recognized as broken DNA [2,3]. Telomere is a typical fragile site, which bears very high loads of replication stress. If the replication stress cannot be resolved properly, the genome would be unstable. Incomplete chromosome ends replication leads to telomere progressive shortening, and then results in telomere dysfunction and replicative senescence [4].

The telomeric repeat-containing RNA, also termed TERRA, is a polymerase II (RNAPII) transcribed RNA, which is conserved throughout eukaryotes [5–7]. TERRA is a kind of lncRNA that transcribed from subtelomeres to telomeres, and contains the subtelomeric regions and telomeric sequences [7]. In human cells, TERRA is heterogeneous in size, ranging between 100 bp and 9 kb [6,8]. It has been speculated that TERRA is prone to form RNA-DNA hybrids with the C-rich telomeric strand, resulting in the displacement of the telomere G strand, giving rise to a structure called R-loop [9–13]. Previous studies have shown that the expression and localization of TERRA is influenced by multiple factors, including telomere length, telomeric chromatin stage, exogenously inflicted DNA damage, ontogenesis, and so on [6,14,15]. In telomerase-negative human cancer cell lines, which maintain telomere lengths by homologous recombination-based alternative lengthening of telomeres (ALT) mechanism, both TERRA levels and telomeric R-loops are upregulated [8,11]. Too many R-loops cause replication fork stalling and collapse, and thus hinder DNA replication genome-wide [16]. For example, depletion of RNase H1 (the enzyme that digests RNA:DNA hybrids) in these cells will lead to enhanced R-loop formation, abundant replicative stress, telomere fragility, and more extra-chromosomal telomere repeat content [11]. Therefore, the expression of TERRA needs delicate regulation.

The RNA binding motif protein encoded on the X chromosome (RBMX), also known as RNA binding protein heterogeneous nuclear ribonucleoprotein G (hnRNP G), belongs to a large family of hnRNPs (A-U), which have a highly conserved RNA-binding domain and lie behind pre-mRNA splicing [17]. It was identified as a paralog of RBMY, a Y chromosome-linked gene, which has been associated with liver cancer activation [18] and responsible for spermatogenesis [19,20]. RBMX is originally recognized as a part of spliceosome, and participate in the function of alternative splicing [17,21,22]. In recent years, RBMX is rediscovered to function in cohesion of sister chromatids, DNA damage repair, and the assembling of higher-order ribonucleoprotein complex for promoting genomic stability [23–25]. It has been recently reported that RBMX is involved in the activation of ATR to maintain genome stability during replication [26]. Here, we discovered that RBMX interacts with telomeric TERRA and plays a novel role in the regulation of TERRA degradation and the formation of telomeric R-loop to maintain telomere stability.

## Results

### RBMX deficiency increases telomeric replication stress

**Fig 1:**
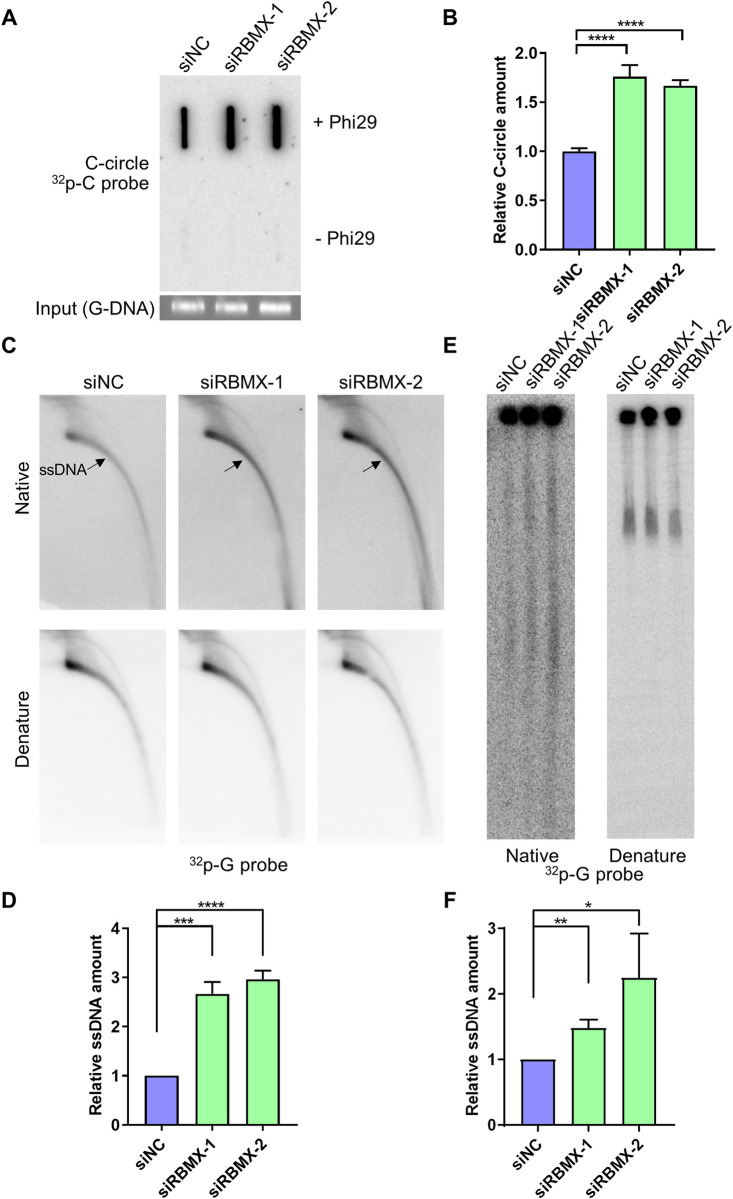
RBMX deficiency increases telomere replication stress in U2OS cells. (**A**) C-circle increases in RBMX-depleted U2OS cells. Cells were transfected with siRNAs for 72 h, and total DNA was extracted. C-circle was amplified by Φ29 and determined by slot blot using telomeric C-probe, with genomic DNA as loading control, and the group without Φ29 as negative control. (**B**) Quantification of (A). The amount of C-circle was calculated as C-circle intensity/G-DNA intensity, and then normalized to the siNC group. (**C**) C-rich ssDNA increases in RBMX-depleted U2OS cells. Genomic DNA was purified from RBMX-depleted U2OS cells and subjected to 2D gel electrophoresis and hybridization using ^32^P-G probe under native or denaturing conditions. The arrow indicates ssDNA signal. (**D**) Quantification of (C). The amount of relative ssDNA was calculated, and then normalized to the siNC group. (**E**) C-rich ssDNA increases in RBMX-depleted U2OS cells. RBMX-depleted U2OS cells were collected and analyzed by contrast-field gel electrophoresis (CFGE). Telomeric fragments were detected by hybridization of ^32^P-G probe under native or denaturing conditions. (**F**) Quantification of (E). The amount of relative ssDNA was calculated, and then normalized to the siNC group. Error bars, standard deviations from ≥3 biological replicates. p values, two-tailed Student’s t test (*p<0.05, **p <0.01, ***p <0.001, ****P <0.0001).

To explore the possible role of RBMX at telomeric region, RBMX was knocked down in human U2OS cells, an ALT cell line that is characterized by a high level of endogenous DNA replication stress [27]. RBMX depleted U2OS cells had much higher level of C-rich extrachromosomal circles (C-circles) than control cells as displayed by Φ29 DNA polymerase based C-circle assay (Fig 1A and 1B). In addition, the C-rich single-stranded DNA (ssDNA) is increased significantly after RBMX depletion as detected by two-dimensional agarose gel electrophoresis assay (Fig 1C and 1D). We further detected the C-rich ssDNA by embedding cells in the agarose and lysing *in situ*, thus only DNA fragments but not intact genomic DNA can be released into the gel during electrophoresis, and the C-rich ssDNA was detected using G-rich probe under native condition. The result showed that C-rich ssDNA increased after RBMX depletion whereas the total signal detected under denatured condition did not change (Fig 1E and 1F). Both C-circles and C-rich ssDNA are by-products of telomere replication in ALT cells with unknown function, and their production is associated with replication fork collapse [28]. Hence, it seems that RBMX deficiency increases telomeric replication stress in ALT cells and leads to accumulation of C-circles and C-rich ssDNA.

**Fig 2:**
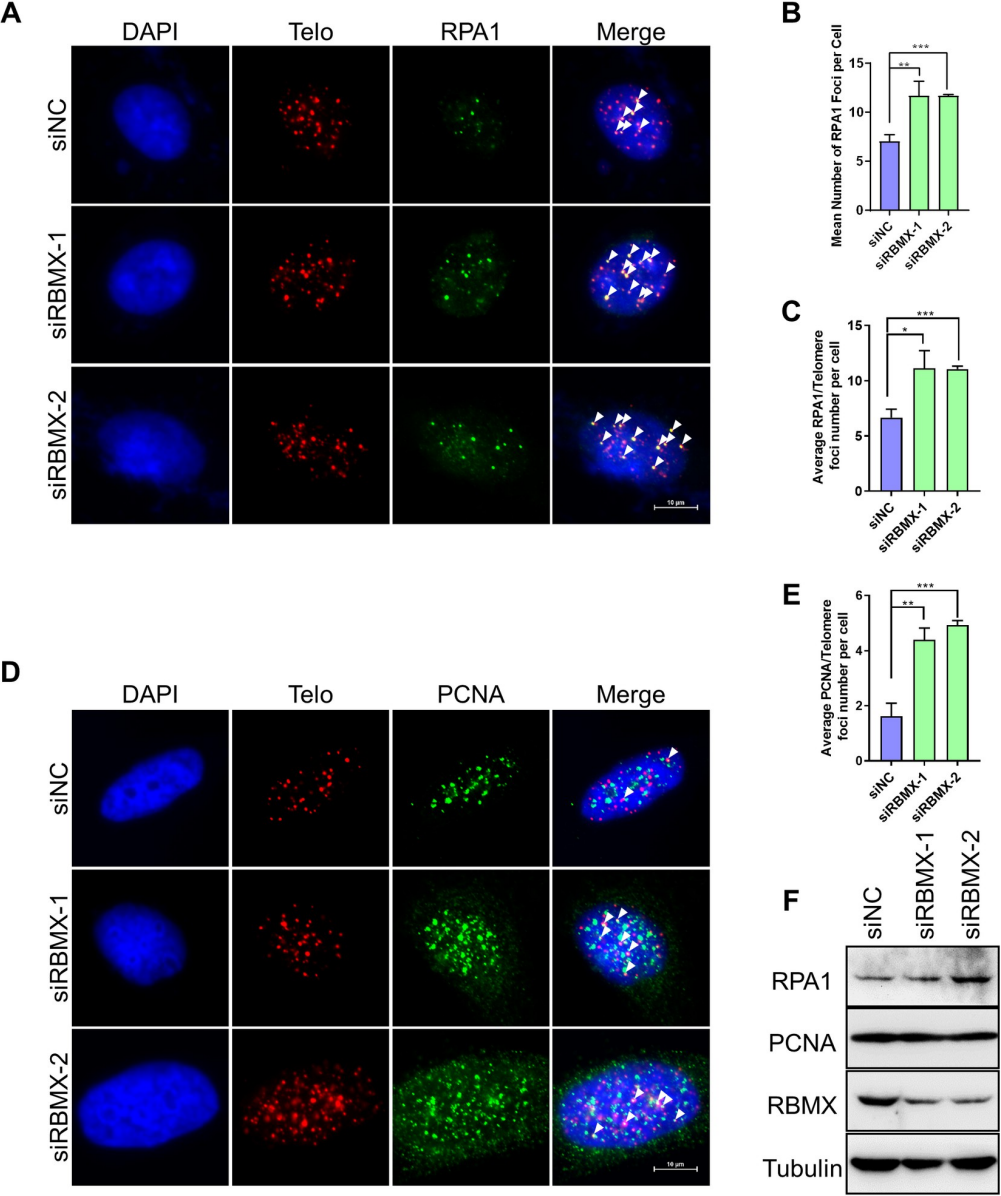
Depletion of RBMX causes replication defects at telomeres. (**A**) RPA1 foci at telomeres increase in RBMX-depleted U2OS cells. RPA1 and telomeres were detected by IF-FISH using anti-RPA1 antibody and G probe, respectively. Scale bars, 10μm. Arrows indicate co-localization events. (**B**) Quantification of (A). The mean numbers of RPA1 foci per cell were counted (n ≥100 cells × 3 repeats). (**C**) Quantification of (A). The mean numbers of RPA1 foci co-localized with telomeres were counted (n ≥100 cells × 3 repeats). (**D**) PCNA foci at telomeres increase in RBMX-depleted U2OS cells. PCNA and telomeres were detected by IF-FISH using anti-PCNA antibody and G probe, respectively. Scale bars, 10μm. Arrows indicate co-localization events. (**E**) Quantification of (D). The mean numbers of PCNA foci co-localized with telomeres were counted (n ≥100 cells × 3 repeats). (**F**) Western blot analysis of RPA1, RBMX and PCNA in U2OS cells transfected with siNC or siRBMX-1/2. p values, two-tailed Student’s t test (*p<0.05, **p <0.01, ***p <0.001).

To validate this hypothesis, we examined two key markers of DNA replication, RPA and PCNA. RPA is the replication protein A that binds to and stabilizes ssDNA after DNA unwound during replication or DNA damage repair [29], whereas PCNA is the proliferating cell nuclear antigen that tethers polymerases and slides them along the double stranded DNA helix [30]. Upon RBMX knockdown, both of the overall RPA1 foci number and the foci number on telomere are increased (Fig 2A–2C). In addition, the number of PCNA foci on telomere also increased significantly (Fig 2D–2E). The total PCNA level did not change, whereas increased RPA1 level was detected (Fig 2F). These results indicate that telomere replication stress boosted after RBMX knockdown.

### RBMX binds TERRA directly and regulates TERRA stability

**Fig 3:**
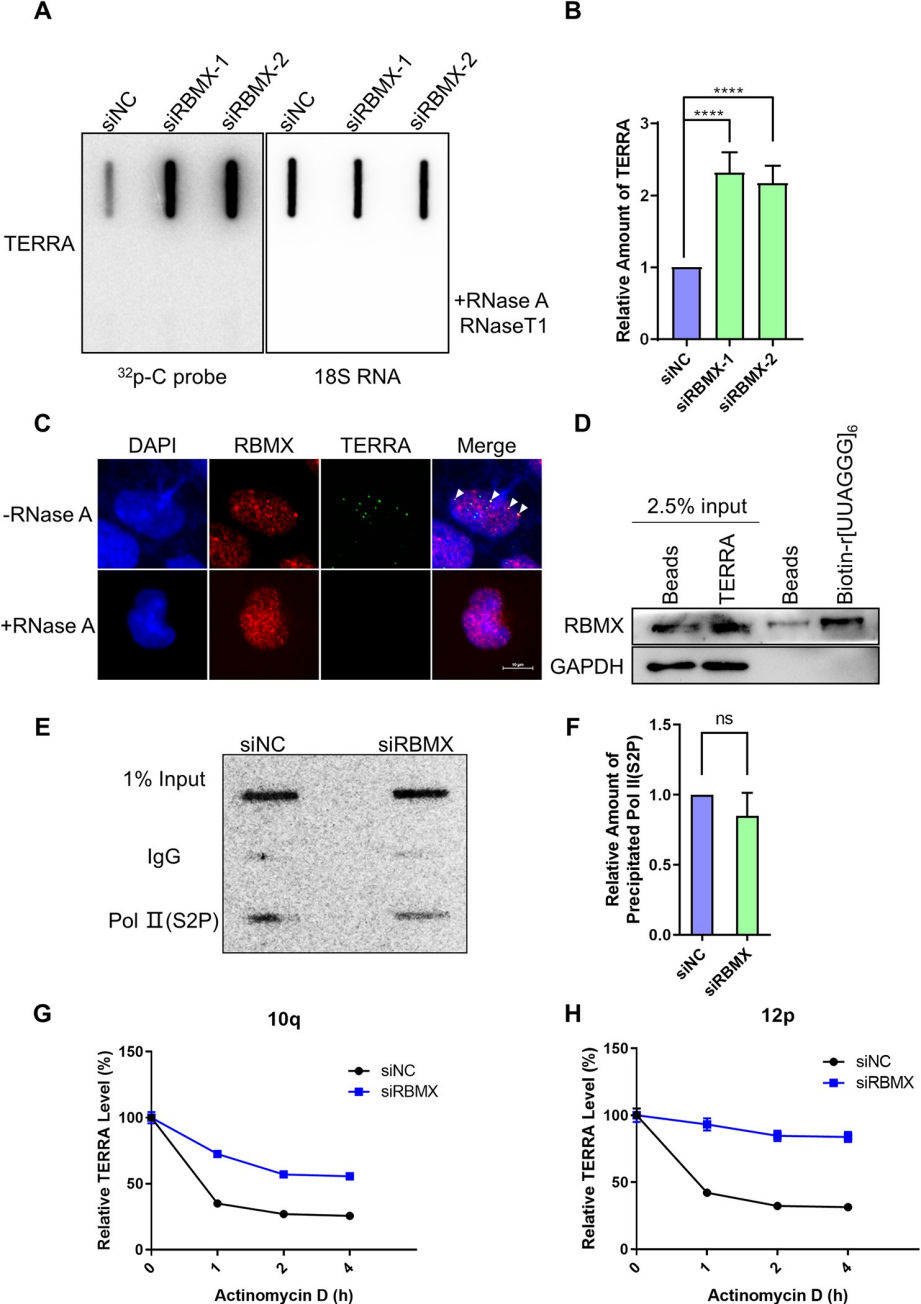
RBMX interacts with TERRA directly and regulates TERRA stability. (**A**) TERRA increases in RBMX-depleted U2OS cells. Slot blot was performed to determine the TERRA level, the RNA samples were treated with or without RNase A and RNase T1, with 18S RNA as loading control. (**B**) Quantification of (A). The amount of TERRA was calculated as TERRA intensity/18S RNA intensity, and then normalized to the siNC group. (**C**) RBMX foci co-localize with telomeres in U2OS cells. Arrows indicate co-localization events. (**D**) RBMX binds to (UUAGGG)_n_ RNA oligos. Biotin-labeled (UUAGGG)_n_ RNA oligos were incubated with U2OS nuclear extract and RNA pulldown was performed using Streptavidin beads. The products were determined by western blot. (**E**) Active RNA Pol II on telomeres does not change in RBMX-depleted U2OS cells. ChIP was performed using Ser2-phosphorylated Pol II (Pol II S2P) antibody, and telomeres were detected by slot blot using C probe. (**F**) Quantification of (E). The relative intensity of precipitated telomeric DNA signals. (**G, H**) TERRA degrades slowly in RBMX-depleted U2OS cells. Transcription was inhibited by 5 g/ml Actinomycin D in RBMX-depleted and control U2OS cells. The TERRA transcribed from 10q chromosome (G) and 12p chromosome (H) were determined by RT-qPCR at the indicated time points. GAPDH were used for normalization. Error bars, standard deviations from ≥3 biological replicates. p values, two-tailed Student’s t test (****P <0.0001).

Considering that RBMX is an RNA binding protein, and that TERRA is a kind of lncRNA that regulates the telomere structure and stability, it is very likely that RBMX impacts telomere integrity through interacting with TERRA. To test this possibility, we firstly examined total TERRA level in both control and RBMX-depleted U2OS cells, and observed that TERRA increased significantly after RBMX knockdown (Fig 3A and 3B). The same phenomenon was observed in telomerase positive HeLa cells (S1 Fig). Next, the RBMX immunofluorescence and TERRA RNA-FISH showed that RBMX foci co-localized with TERRA foci, which could be eliminated upon RNase A digestion (Fig 3C). Subsequently, we performed RNA-pulldown assay to check whether RBMX binds to TERRA directly. Biotin labeled RNA oligonucleotides containing 6 repeats of UUAGGG (TERRA) were incubated with nuclear extracts of U2OS cells, and then recovered using streptavidin coated magnetic beads. Eluates were processed by western blot using RBMX antibody, and the RBMX was successfully pulled down by synthetic TERRA (Fig 3D), indicating that RBMX binds to TERRA directly.

To figure out how RBMX influences TERRA levels, ChIP assay was performed using the antibody against Ser2-phosphorylated Pol II (Pol II-S2P), which is considered as a marker for active or elongating Pol II [31], to detect whether RBMX regulates the transcription activity of telomeres. The products were determined by slot blot and hybridized with telomeric probe. It is demonstrated that there was no change of Pol II-S2P level at telomeres regardless of whether RBMX was depleted or not (Fig 3E and 3F), suggesting that RBMX does not regulate TERRA transcription.

Hence, further experiments were performed to investigate TERRA stability in the absence of RBMX. Both control and RBMX-depleted U2OS cells were treated with actinomycin D to block de novo transcription. Then TERRA transcripts from 10q and 12p chromosome ends were quantified at indicated time points using qRT-PCR with subtelomere-specific PCR primers [32] and normalized to GAPDH. It’s obvious that TERRA level decayed to less than 50% within 1 hour in control group, while TERRA degraded very slowly when RBMX was silenced (Fig 3G and 3H). This indicated that the degradation of TERRA is regulated by RBMX.

**Fig 4:**
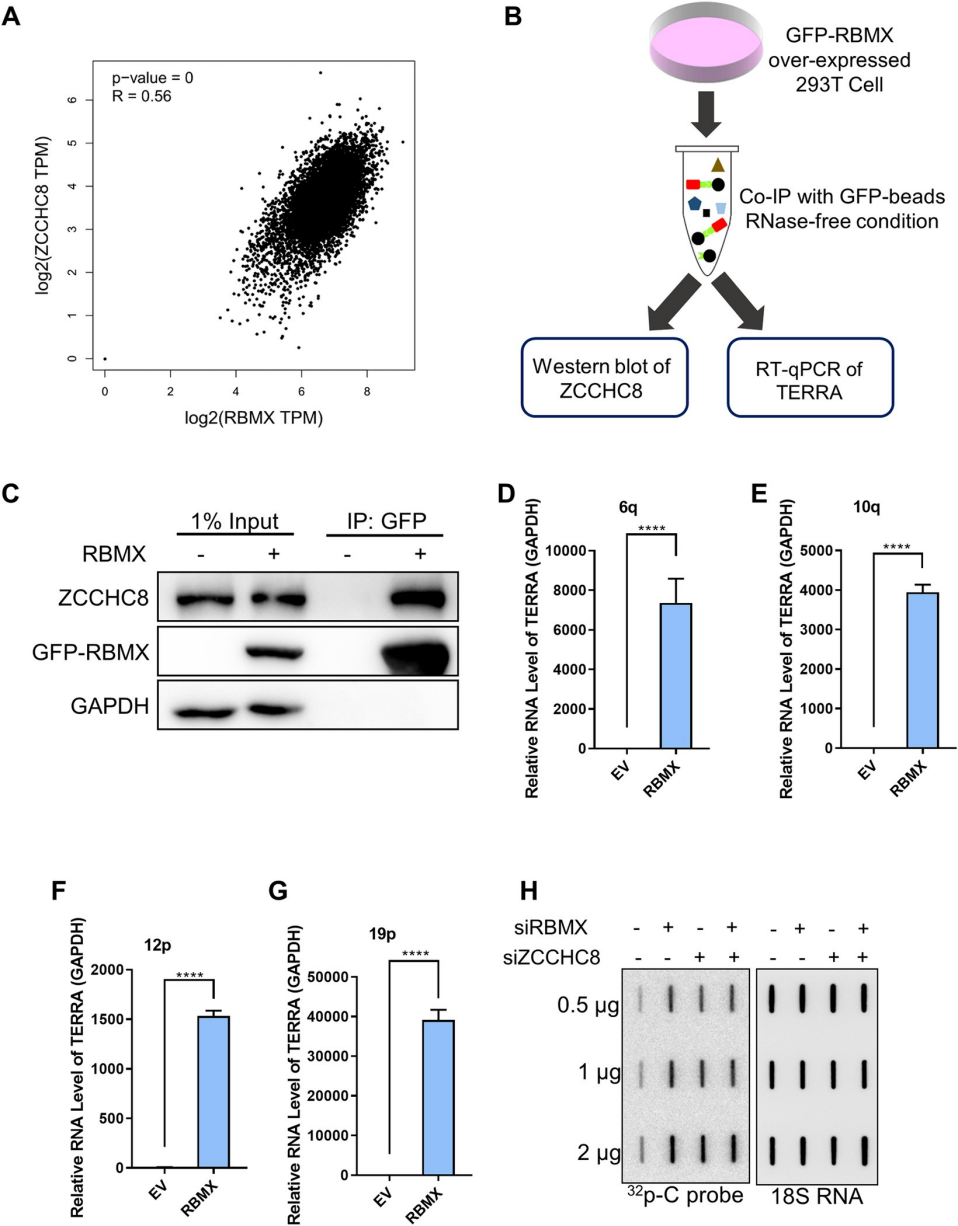
RBMX regulates TERRA level by interacting with ZCCHC8. (**A**) Pair-wise Pearson correlation between RBMX and ZCCHC8 mRNA levels. The website tool GEPIA was used for correlation analysis, with the gene expression data (TPM value) from TCGA tumor (n>1000). (**B-G**) RBMX binds to ZCCHC8 and TERRA in 293T cells. (B) Schematic diagram. Interaction between ectopically expressed GFP-tagged RBMX, endogenous ZCCHC8 and TERRA was detected by co-immunoprecipitation with anti-GFP antibody in RNase-free condition. Half of the co-IP eluates were used for protein detection by western blot (C), while the other half were used for TERRA detection by RT-qPCR, including TERRA transcribed from 6q, 10q, 12p, and 19p chromosomes (D-G). (**H**) TERRA increases in U2OS cells transfected with indicated siRNAs. Slot blot was performed to determine the TERRA level, with 18S RNA as loading control. Error bars, standard deviations from ≥3 biological replicates. p values, two-tailed Student’s t test (****P <0.0001).

The turnover of nuclear RNA is subject to surveillance of the nuclear exosome which contains multi-subunit ribonucleolytic complex that degrades RNA [33]. Interestingly, it is reported that RBMX was present in exosomes isolated from ovarian cancer cells and endothelial cells [34,35]. Then, we wonder whether RBMX targeted TERRA to nuclear exosome for degradation. To explore this possibility, we analyzed the correlation between RBMX and ZCCHC8 mRNA levels. ZCCHC8 is the key component of the nuclear exosome targeting (NEXT) complex and cooperates with other members of NEXT to recognize and deliver transcripts to the exosome for decay [36–38]. By pair-wise Pearson correlation analysis using GEPIA website tool with tumor data from TCGA database [39], we found that the mRNA expression level of ZCCHC8 is positively correlate with RBMX level (Fig 4A), suggesting that RBMX might regulate TERRA degradation by interacting with ZCCHC8.

To validate this possibility, we performed RBMX co-immunoprecipitation in an RNase-free condition and the product was detected by western blot for proteins and by RT-qPCR for TERRA respectively to examine the physical interaction between RBMX, ZCCHC8 and TERRA (Fig 4B). By the Co-IP-qPCR assay, we observed that ZCCHC8 is co-precipitated with ectopically expressed GFP-tagged RBMX in 293T cells (Fig 4C), and TERRA that transcribed from different chromosome ends (6q-TERRA, 10q-TERRA, 12p-TERRA, 19p-TERRA) were also enriched by the GFP-tagged RBMX (Fig 4D–4G). These results revealed that RBMX binds to ZCCHC8 and TERRA simultaneously. In addition, we found that RBMX-ZCCHC8 double knockdown did not further elevate the TERRA level which is already increased by RBMX or ZCCHC8 knockdown alone (Figs S2A and 4H), suggesting RBMX and ZCCHC8 regulate TERRA level in a single pathway.

### Depletion of RBMX enhances R-loop formation at telomeres

**Fig 5:**
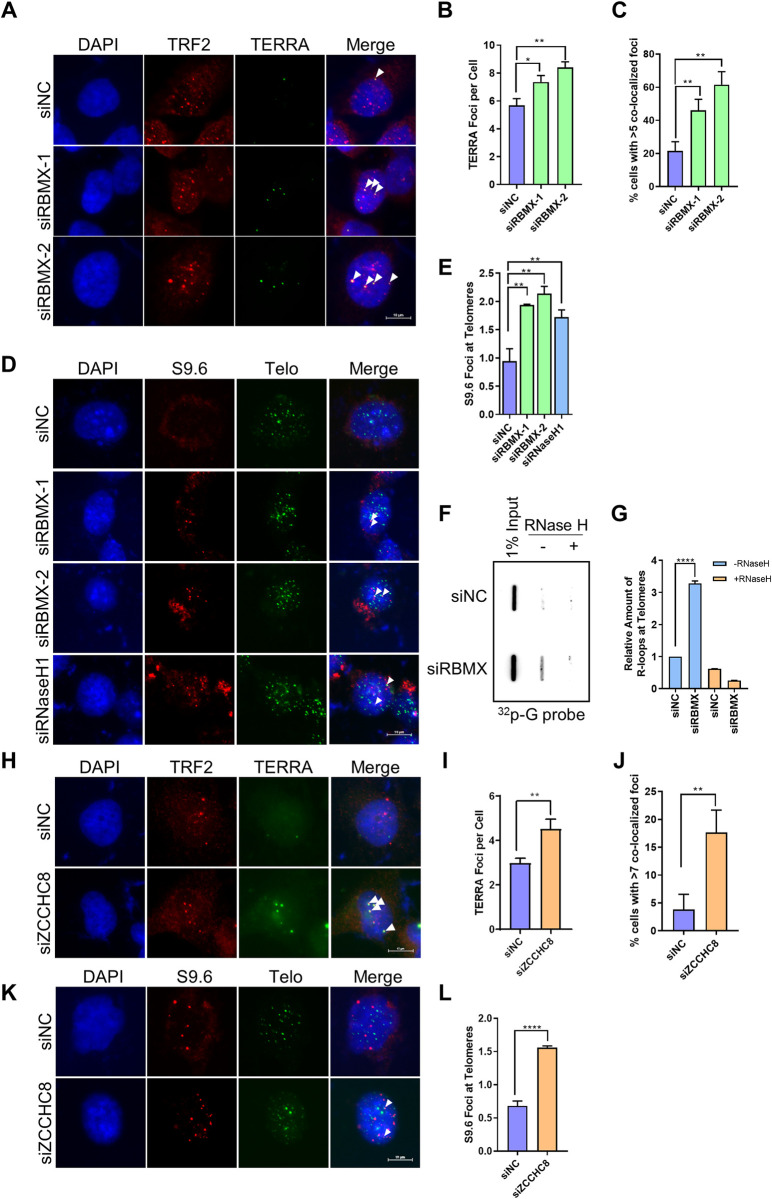
RBMX deficiency stimulates R-loop formation at telomeres. (**A**) TERRA foci at telomeres increases in RBMX-depleted U2OS cells. TERRA and TRF2 were detected by IF-FISH using C probe and anti-TRF2 antibody, respectively. Scale bars, 10 μm. Arrows indicate co-localization events. (**B**) Quantification of (A). The mean numbers of TERRA foci per cell were counted (n ≥100 cells × 3 repeats). (**C**) Quantification of (A). The percentage of cells with more than 5 TERRA foci co-localized with telomeres per nucleus were calculated (n ≥100 cells × 3 repeats). (**D**) R-loop foci at telomeres increases in RBMX-depleted U2OS cells. R-loops and telomeres were detected by anti-S9.6 antibody and C probe, respectively. Scale bars, 10 μm. Arrows indicate co-localization events. (**E**) Quantification of (D). The mean numbers of R-loop foci co-localized with telomeres were counted (n ≥100 cells × 3 repeats). (**F**) DRIP analysis of R-loops at telomeres in control and RBMX deficient cells. S9.6 antibody was used to pull-down R-loops containing DNA, which was then subjected to slot blot using G probe. Samples digested with RNase H was used as a control. (**G**) Quantification of (F). The relative amount of telomeric R-loops was determined. (**H**) TERRA foci at telomeres increases in ZCCHC8-depleted U2OS cells. TERRA and TRF2 were detected by IF-FISH using C probe and anti-TRF2 antibody, respectively. Scale bars, 10μm. Arrows indicate co-localization events. (**I**) Quantification of (H). The mean numbers of TERRA foci per cell were counted (n ≥100 cells × 3 repeats). (**J**) Quantification of (H). The percentage of cells with more than 7 TERRA foci co-localized with telomeres per nucleus were calculated (n ≥100 cells × 3 repeats). (**K**) R-loop foci at telomeres increases in ZCCHC8-depleted U2OS cells. R-loops and telomeres were detected by IF-FISH using anti-S9.6 antibody and C probe, respectively. Scale bars, 10 μm. Arrows indicate co-localization events. (**L**) Quantification of (K). The mean numbers of R-loop foci co-localized with telomeres were counted (n ≥100 cells × 3 repeats). p values, two-tailed Student’s t test (*P <0.05, **P <0.01, ****P <0.0001).

TERRA regulates telomere stability. It impairs telomere replication, through forming R-loop with the telomeric C-rich DNA strand, which is also the templates for both TERRA transcription and leading-strand replication [11]. Hence, we wished to know whether RBMX affect R-loop formation in U2OS cells. Firstly, we checked the localization of TERRA on telomere and observed that depletion of RBMX led to significant increase of TERRA foci both totally and on telomere (represent as TRF2) (Fig 5A–5C). Then, we detected the R-loop directly using S9.6, a specific antibody that recognizes RNA-DNA hybrids, and observed more R-loop signals co-localized to telomeres after RBMX depletion (Fig 5D–5E). This result is similar to the previously report that RNase H1 knockdown increase the abundance of telomeric R-loops [11] (Figs 5D–5E and S2B). In addition, we performed DNA-RNA immunoprecipitation using S9.6 antibody (DRIP assay) followed by hybridization with telomeric probe and the result showed that the amount of telomere precipitated by S9.6 increased after RBMX depletion, and the signals are sensitive to RNase H digestion (Fig 5F–5G). What is more, knockdown of ZCCHC8 also led to not only increased TERRA level and TRF2-TERRA co-localization signals (Figs S2C and 5H–5J), but also increased abundance of telomeric R-loops (Fig 5K–5L). Together with Fig 4, these results indicate that RBMX regulates TERRA and telomeric R-loop level by cooperating with ZCCHC8 in U2OS cells.

### Depletion of RBMX leads to telomere instability

**Fig 6:**
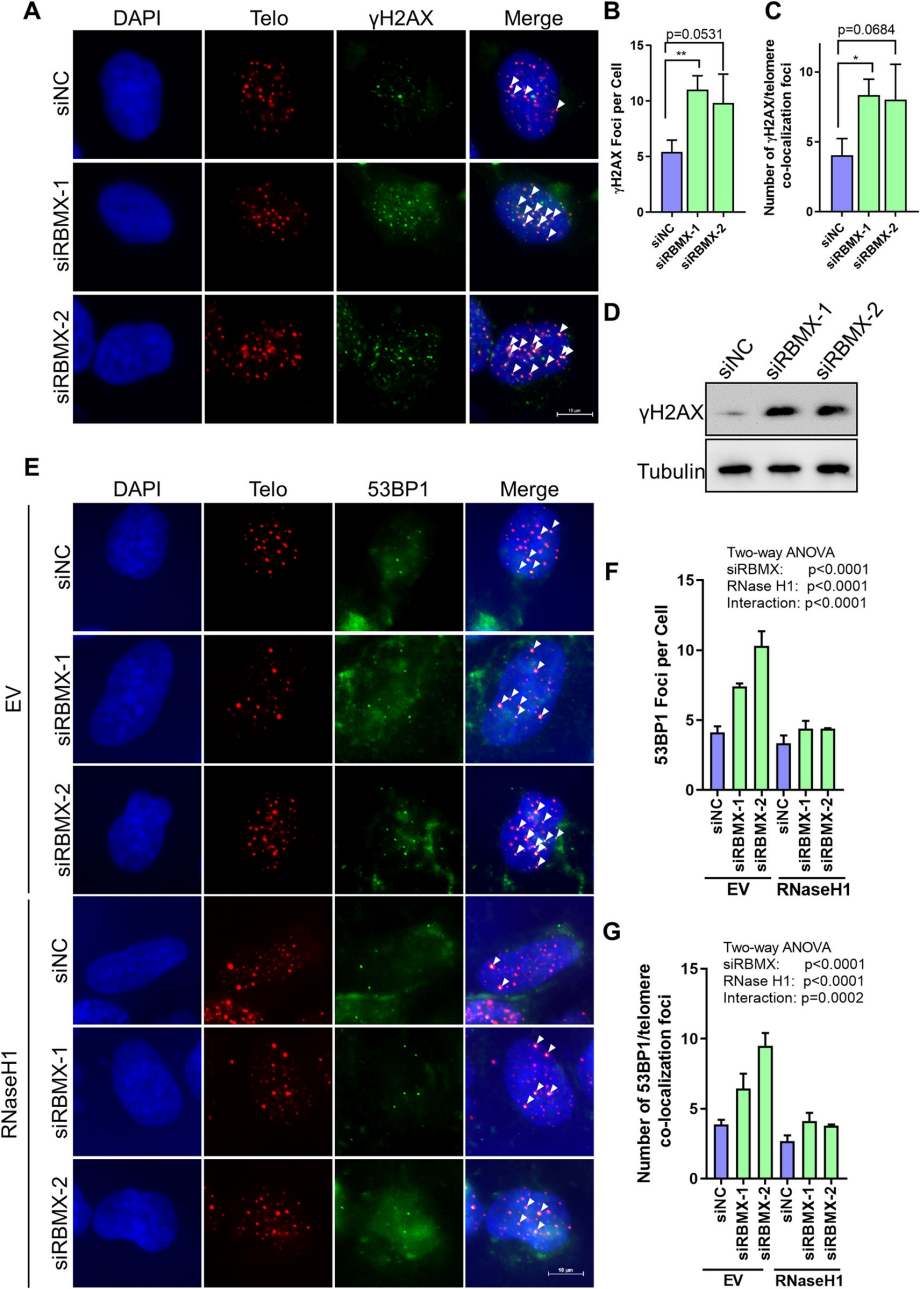
Depletion of RBMX increases telomeric DNA damage. (**A**) γH2AX foci at telomeres increases in RBMX-depleted U2OS cells. γH2AX and telomeres were detected by anti-γH2AX antibody and G probe, respectively. Scale bars, 10μm. (**B**) Quantification of (A). The mean numbers of γH2AX foci per cell were counted (n ≥100 cells × 3 repeats). (**C**) Quantification of (A). The mean numbers of γH2AX foci co-localized with telomeres were counted (n ≥100 cells × 3 repeats). (**D**) Western blot analysis of γH2AX in U2OS cells transfected with siNC or siRBMX-1/2. (**E**) 53BP1 foci at telomeres increases in RBMX-depleted U2OS cells, and can be rescued by over-expression of RNase H1. 53BP1 and telomeres were detected by anti-53BP1 antibody and G probe, respectively. Scale bars, 10μm. (**F**) Quantification of (E). The mean numbers of 53BP1 foci per cell were counted (n ≥100 cells × 3 repeats). (**G**) Quantification of (E). The mean numbers of 53BP1 foci co-localized with telomeres were counted (n ≥100 cells × 3 repeats). For panel B&C, two-tailed Student’s t test was used to determine the statistical significance (*p<0.05, **p <0.01). For panel F&G, the Two-way ANOVA was performed.

The formation of TERRA-telomere R-loop structures appear as obstacles during DNA replication, leading to both replicative stress and DNA damage at telomeres. Therefore, we visualized the DNA damage foci on telomeres by immuno-staining and fluorescence *in situ* hybridization (IF-FISH), using antibody against γH2AX/53BP1 (well-known DNA damage markers) and a telomeric probe. As expected, the total number of γH2AX/53BP1 foci or foci on telomeres increased significantly when RBMX was depleted (Fig 6A–6C and 6E–6G), and western blot also showed that the γH2AX expression level is increased (Fig 6D). Meanwhile, over-expression of Flag-RNase H1 in RBMX depleted cells successfully reduced the 53BP1 foci close to control group (Figs S2D and 6E–6G). Therefore, loss of RBMX leads to increased telomeric DNA damages because of increased telomeric R-loops in U2OS cells.

**Fig 7:**
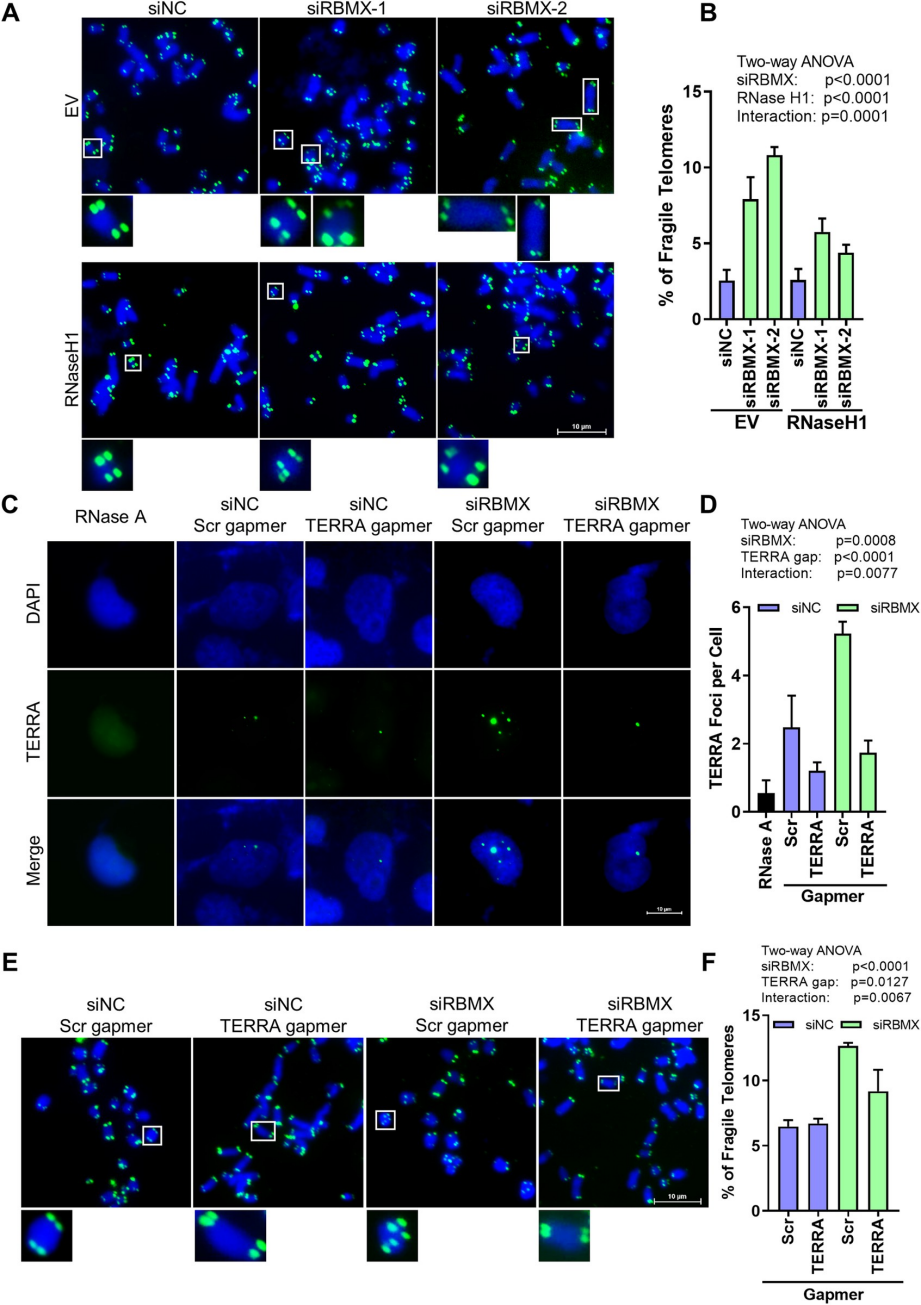
RBMX deficiency leads to telomere instability. (**A**) Metaphase telomere FISH detection of fragile telomere signals at the end of chromosomes in RBMX-deficient U2OS cells co-transfected with Flag-RNase H1. The fragile telomere signals increase in RBMX-depleted U2OS cells, and can be rescued by over-expression of RNase H1. (**B**) Quantification of (A). The percentage of chromosomes with multiple telomeres signals were calculated. For each group, 1000 or more chromosomes were examined. (**C**) RNA FISH detection of TERRA in siNC or siRBMX transfected U2OS cells treated with scrambled LNA gapmer (Scr gapmer) or TERRA targeting LNA gapmer (TERRA gapmer). Cells were treated with gapmers for 24 h prior to analysis. (**D**) Quantification of (C). The average number of TERRA foci per cell was determined (n ≥100 cells × 3 repeats). (**E**) Metaphase telomere FISH detection of fragile telomere signals at ends of chromosomes. siNC or siRBMX transfected U2OS cells were treated with scrambled LNA gapmer or TERRA targeting LNA gapmer for 24 h, then treated with Colchicine for 6 h, and subjected to Metaphase FISH. (**F**) Quantification of (E). The percentage of chromosomes with multiple telomeres signals were calculated. For each group, 1000 or more chromosomes were examined. Two-way ANOVA was used to determine the statistical significance.

It has been reported that accumulated replication stress or DNA damages at telomeres would lead to fragile telomeres [40], so we performed metaphase telomere FISH to inspect the fragile telomeres after RBMX depletion. Increased number of multiple telomere signals (MTS) were detected in RBMX-depleted U2OS and HeLa cells (Figs 7A, 7B, S3A and S3B), revealing that RBMX depletion induces fragile telomeres. Meanwhile, overexpression of Flag-RNase H1 in RBMX deficient cells reduced the elevated amount of fragile telomere significantly (Fig 7A and 7B), supporting the idea that R-loops are the source of fragile telomeres. To further validate this conclusion, we knocked down TERRA (Fig 7C and 7D) using a locked nucleic acid (LNA) gapmer against TERRA [41], and the amount of fragile telomeres decreased significantly in RBMX depleted cells (Fig 7E and 7F), displaying that the telomere instability caused by RBMX depletion can be reversed by TERRA subtraction. Therefore, the RBMX affects telomere integrity by regulating TERRA degradation and R-loop formation at telomeres.

Considering that RBMX activates ATR through recruiting TopBP1 to ssDNA in the circumstances of replication stress [26], it is possible that RBMX depletion leads to telomere instability through inhibiting ATR activation. To test it, we treated cells with ATR kinase inhibitor AZD6738 in combination with RBMX knockdown. The result showed that AZD6738 treatment alone displays similar effect to RBMX depletion, and simultaneously inhibiting ATR and knocking down RBMX further elevated the amount of fragile telomeres (S3C and S3D Fig). Together with Fig 7, these results suggest that RBMX depletion induces telomere instability mainly by elevating TERRA/R-loop level and its role in ATR activation maybe contribute to telomere instability, but with limited effect.

## Discussion

In this study, we demonstrated that RNA binding protein RBMX plays a central role in TERRA degradation, and therefore suppresses telomeric R-loop formation to maintain telomere stability. We observed that increase of TERRA level in RBMX-depleted U2OS cells results in formation of R-loops that impede telomere replication and impairs telomere stability. In addition, we discovered a previously unrecognized function of RBMX: regulating TERRA stability by interacting with both TERRA and ZCCHC8. Our results open new avenues in understanding the importance of RNA binding proteins in maintaining telomere integrity.

### RBMX regulates the stability of TERRA

It has been previously reported that TERRA plays a physiologically relevant role in telomere function [42,43], and it is important to maintain homeostasis of TERRA level. Since TERRA formed R-loop contributes to telomere homologous recombination (HR) [7,9,11,13,43,44], the telomere DNA damage repair or alternative lengthening of telomere, both of which are based on HR, will be disturbed if TERRA is insufficient. Our recent work showed that TERRA is more stable with m6A modification, and downregulated TERRA caused by METTL3 deficient leads to telomere instability and telomere shortening in ALT cells [45]. On the other hand, excessive TERRA in the cell forms redundant telomeric R-loops, which impede the progression of telomere replication and result in fragile telomeres [46]. It also reported that abnormally elevated TERRA level is correlated with human immunodeficiency, centromeric region instability, and facial anomalies syndrome with telomere aberrations such as telomere free-ends [47]. In this study, we uncovered that RBMX plays an important role in restraining TERRA expression level, and maintaining optimal telomeric R-loop numbers. We observed that loss of RBMX results in significant increase of TERRA level, and depletion of RBMX extends the half-lives of TERRA transcripts (Fig 3). The interaction between RBMX, TERRA and ZCCHC8 suggesting that RBMX may accelerate the degradation of TERRA through targeting TERRA to NEXT complex (Fig 4), which shuttles transcripts to the nuclear exosome for degradation [36]. Hence, this study uncovered that TERRA decays in an RBMX dependent pathway. Since RBMX is an RNA-binding protein, it is possible that it also binds to and regulates the degradation of other RNA in the nuclear. This is an interesting hypothesis worthy of further investigation.

### The new function of RBMX in telomere stability

As an RNA binding protein, RBMX has been reported to implicate in different biological processes, including but not limited to alternative splicing, transcription control, and genomic stability regulation [48]. It regulates genomic stability through kinds of way, such as chromosome biology and DNA damage repair. In terms of chromosome biology, RBMX regulates the centromere in the cell cycle, and its depletion causes premature loss of sister chromatid cohesion [24] and accumulation of pre-metaphase HeLa cells, with a failure of chromosome alignment on the metaphase plate [49]. In terms of DNA damage repair, RBMX is reported to accumulate at DNA lesions and promote homologous recombination and its depletion sensitizes cells to DNA damage [23]. What’s more, RBMX has been shown to interact with the long non-coding RNA NORAD (non-coding RNA activated by DNA damage) and regulate DNA replication and DNA damage repair [25]. Our previous research discovered that RBMX is a key factor to maintain genome stability during replication by activating ATR [26]. Here, we uncovered the new function of RBMX in maintaining telomere stability, which is a key part of genomic stability. We found that absence of RBMX leads to increased telomere replication stress (Figs 1 and 2), R-loops (Fig 5) and fragile telomeres (Figs 6 and 7). The fragile telomeres can be rescued by TERRA knockdown (Fig 7), and enhanced by ATR inhibitor (S3 Fig), supporting the idea that RBMX regulates telomere stability mainly by regulating TERRA level and enhancing R-loop formation at telomeres. The ATR pathway contributes partially to RBMX deficiency induced telomere instability.

Altogether, these studies reveal that RBMX is a multifunctional protein that maintains genomic stability in various ways.

### Potential role of RBMX in cancer therapy

Genomic instability is one of the leading causes of carcinogenesis. Hence, it is possible that RBMX is a potential tumor suppressor. Actually, there are several lines of evidence implicating the role of RBMX in cancer. In genome sequencing in lung cancer patients, mutations truncating the *RBMX* gene are identified, probably induced by tobacco smoke [50]. In the absence of *RBMX* gene, some thyroid cancers (papillary thyroid carcinoma) obtain vemurafenib resistance [51]. In endometrial cancer, increased *RBMX* level is connected with a favorable outcome [52]. Consistent with this, *RBMX* may prevent the development of oral tumors [53]. Here, we found that RBMX deficiency results in telomere replication stress and telomere DNA damage, causing telomere instability and dysfunction (Figs 1, 6 and 7), which may contribute to the pathogenesis of some types of tumors. Thus, RBMX may be a promising target for cancer therapy.

## Materials and methods

### Cell culture and transfection

U2OS, HeLa, and HEK293T cells were obtained from American Type Culture Collection (Manassas, VA, USA). Cells were grown in Dulbecco’s modified Eagle’s medium (DMEM; GIBCO) supplemented with 10% fetal bovine serum (FBS; GIBCO) and 1% penicillin/streptomycin (GIBCO) at 37°C and 5% CO_2_. siRNA was transfected into cells using Lipofectamine RNAiMAX Transfection Reagent (Thermo Fisher Scientific) according to the manufacturer’s instructions. The plasmids were transfected into cells using Lipofectamine 3000 Transfection Reagent (Thermo Fisher Scientific).

Sequences of the various siRNAs used in the study are: siNC (Negative Control): 5′-UUCUCCGAACGUGUCACGUdTdT-3′; siRBMX-1: 5’-UGCUUCAAGAGCUUUCUCAdTdT-3’; siRBMX-2: 5’-UUCAUCAAGAGUACUUCCAdTdT-3’; siZCCHC8: 5’-GGAAUGUACCUCAGGAUAAdTdT-3’; siRNaseH1: 5’-ACCAAAGAGCGGAAAUUCAUGdTdT-3’.

All the cell lines were identified by standardized short tandem repeat analysis. Mycoplasma was regularly examined during cell culturing, and no contamination occurred during this study.

### Native/denatured in-gel hybridization

In gel hybridization analysis of the DNA samples was performed as follows [54]: The gel was dried for more than 1h at room temperature. For native in-gel hybridization, gels were hybridized in hybridization buffer (10 × Denhard’s buffer, 2μg/mL sonicated Escherichia coli DNA, 0.5% SDS and 5 × SSC) with ^32^P-labled C-/G-rich telomeric probe. The probes were prepared as described previously [55]. For denatured in-gel hybridization, gels were denatured with 0.5 M NaOH, neutralized with 1 M Tris-HCl (pH 8.0), and then followed by the native hybridization procedure. The gels were hybridized overnight at 42°C with ^32^P-labled C-/G-rich telomeric probe in the hybridization buffer. After hybridization, the gel was washed for 2 times in wash buffer I (0.1% SDS, 4 × SSC), and 2 times in wash buffer II (0.1% SDS, 2 × SSC). Then exposed to a PhosphorImager screen (GE Healthcare Life Science) and scanned on Typhoon imager (GE Healthcare Life Science). Blots were quantified and calculated based on the intensity of signal detected. Image Quant software was used for data analysis.

### C-circle assay

C-circle assay was performed as described previously [56]. For U2OS cells, 30 ng of genomic DNA was used for the C-circle assay. Each assay was repeated for 3 times to obtain the quantitative result. 1 μL of each fraction was incubated in 40 μL reaction containing 19 μL ddH_2_O and 20 μL C-circle amplification master buffer (1mM dATP, dGTP and dTTP each, 0.2 mg/mL BSA, 0.1% Tween 20, 1× Φ29 Buffer and 7.5U Φ29 DNA polymerase (Thermo Fisher)) for 8h at 30°C, and then subjected to slot blot and hybridized with ^32^P-labled C-rich telomeric probe.

### Two-dimensional (2D) gel electrophoresis

Two-dimensional (2D) gel electrophoresis was performed as previously described [57,58]. 10 μg genomic DNA was digested overnight at 37°C with restriction enzyme RsaI and HinfI (Thermo Scientific) and RNase A (2 μg/mL, TaKaRa), and then loaded onto a 0.4% agarose gel. Electrophoresis was carried out in 1 × TBE at 1 V/cm at room temperature for 12 h. The lane containing DNA was excised from the gel and the TBE buffer was exchanged with 1 × TBE containing 0.3 μg/mL ethidium bromide (EB; Sigma). After that, the gel slice was transferred and cast with 1% agarose gel in 1 × TBE with 0.3 μg/mL EB. The electrophoresis was carried out at 3 V/cm at 4°C for 6 h. In gel hybridization analysis of telomeric DNA was performed as above.

### Constant-field gel electrophoresis (CFGE) of embedded cells

Constant-field gel electrophoresis (CFGE) assay was performed as previously described [59]. Cells were embedded in 50 μL of pre-warmed (45°C) 0.7% agarose, lysed with 0.5% SDS in Tris-HCl and digested with 100 μg/mL RNase A and 250 μg/mL proteinase K overnight at 37°C. Plugs were placed into the wells of a 0.7% agarose gel, and sealed with 0.7% agarose. Gel electrophoresis was performed using 0.7% agarose in 1 × TAE buffer, and carried out at 1 V/cm at room temperature for 8h. In gel hybridization analysis of telomeric DNA was performed as above.

### Immunofluorescence (IF) and immunofluorescence *in situ* hybridization (IF-FISH)

For IF experiments, cells grown on coverslips were fixed in ice-cold methanol for 10 min at -20°C or in 4% paraformaldehyde for 15 min at room temperature, then permeabilized with 0.2% Triton X-100 for 30min and blocked with 5% goat serum for more than 1h. Fixed cells were incubated sequentially with primary antibody (4°C overnight) and fluorescence-labeled second antibody (room temperature, 1h). Coverslips were stained with DAPI (Vector Laboratories), then visualized and analyzed using Nikon fluorescence microscope.

For IF-FISH experiments, following secondary antibody incubation, cells were fixed with 4% paraformaldehyde for 30 min at room temperature and sequentially dehydrated with 75%, 95%, and 100% ethanol. The coverslips were denatured at 85°C for 5 min, then hybridized with PNA probe (TelC-Alexa488 or TelG-Cy3, Panagene) for 2 h at 37°C. The coverslips were washed, stained with DAPI and visualized and analyzed using Nikon fluorescence microscope.

Antibodies used are as follows: 53BP1 (1:2000, NB100-304, Novus Biologicals), γH2AX (1:200, 9718S, Cell Signaling Technology), RPA1 (1:100, sc-28304, Santa Cruz), PCNA (1:400, ab92552, abcam), RBMX (1:250, ab190352, abcam), TRF2 (1:200, 05–521, Merck), S9.6 (1:100, MABE1095, Millipore).

### Western blot

Cells were directly lysed in 2 × SDS loading buffer and boiled for 15 min. Proteins were separated with SDS-PAGE and transferred to PVDF membrane. The following antibodies were incubated with membrane: RBMX (1:1000, ab190352, abcam), γH2AX (1:2000, Cell Signaling Technology, 9718S), RPA1 (1:1000, sc-28304, Santa Cruz), PCNA (1:1000, ab92552, abcam), ZCCHC8 (1:2000, 23374-1-AP, Proteintech), Flag (1:4000, F1804, Sigma Aldrich), GAPDH (1:5000, 60004-1-Ig, Proteintech), Tubulin (1:5000, 66031-1-Ig, Proteintech), HRP-conjugated anti-rabbit or anti-mouse (KPL, Inc) were then used.

### RNA FISH

TERRA RNA FISH assay was carried out as IF-FISH assay procedure described above in an RNase-free condition, without denaturation at 85°C.

### TERRA RNA-pull down

TERRA RNA-pull down assay was performed as previously described [60] with minor modifications. Cells were resuspended in Buffer A (10 mM HEPES [pH 7.9], 1.5 mM MgCl_2_, 10 mM KCl, 0.5 mM DTT, 1 mM PMSF, Proteinase Inhibitor Cocktail, and 0.05% NP-40), incubated on ice for 10 min. Obtained nuclei were pelleted, and then resuspended in Buffer C (20 mM HEPES [pH 7.9], 1.5 mM MgCl_2_, 0.2 mM EDTA, 400 mM NaCl, 0.5 mM DTT, 1 mM PMSF, Proteinase Inhibitor Cocktail, and 25% Glycerol). The nuclei extract was incubated on ice for 20 min and sonicated (10s/10s, 10 times, 2 round). Samples were centrifuged to obtain the supernatant, which contains nuclear proteins. NaCl concentration was diluted with Buffer C diluent (20 mM HEPES [pH 7.9], 1.5 mM MgCl_2_, 0.2 mM EDTA, 0.5 mM DTT, 1 mM PMSF, and Proteinase Inhibitor Cocktail) to reach a final concentration of 150 mM.

The extracts were pre-cleared using Streptavidin MagneSphere Paramagnetic Particles (Promega), and the beads were blocked by 2.5% BSA in PBS. Beads were removed by the magnetic stand, and the nuclear extracts were supplied with RNase inhibitor (TaKaRa), 100 ng/mL yeast RNA, 5 μg/mL Heparin, and TERRA oligo biotin-(UUAGGG)_6_ and Streptavidin MagneSphere Paramagnetic Particles. Samples were incubated for more than 1.5 h at 4°C. Beads were washed for 3 times in Buffer W (20 mM HEPES [pH 7.9], 150 mM NaCl, 0.2 mM EDTA, 20% Glycerol, 0.5 mM DTT, 1mM PMSF, Proteinase Inhibitor Cocktail, RNase inhibitor, 1% NP-40 and 1% sodium deoxycholate). Bound proteins were eluted using 2 × SDS loading buffer, and boiled for 15min. Eluted proteins were separated on an SDS-PAGE gel and subjected to immunoblotting.

### Quantitative real-time PCR

Total RNA was extracted from cells using RNAiso Plus Reagent (9109, Takara). 1.0 μg of total RNA was reverse-transcribed to cDNA using TransScript One-Step gDNA Removal and cDNA Synthesis Kit (AT311-03, TransGen Biotech) with random primers and TERRA-specific primers (RT-CX5, 5’-CCCTAACCCTAACCCTAACCCTAACCCTAA-3’; RT-CX3, 5’-CCCTAACCCTAACCCTAA-3’) according to manufacturer’s instructions. cDNA was used for real-time PCR using 2 × RealStar Fast SYBR qPCR Mix (A301-10, GenStar). GAPDH was used as internal control for all experiments. The following primers were used for amplification: GAPDH-forward: 5’- AGCCACATCGCTCAGACAC -3’; GAPDH -reverse: 5’- GCCCAATACGACCAAATCC -3’; 6q-TERRA forward: 5’-TTCTGACGCTGCACTTGAAC-3’; 6q-TERRA reverse: 5’-TAGTGTGGAAAGCGGGAAAC-3’; 10q-TERRA forward: 5’- GCCTTGCCTTGGGAGAATCT -3’; 10q-TERRA reverse: 5’- AAAGCGGGAAACGAAAAGC -3’; 12p-TERRA forward: 5’- AGTACCACCGAAATCTGT -3’; 12p-TERRA reverse: 5’- GAGTTGCGTTCTCTTCAG -3’; 19p-TERRA forward: 5’-TTCAGAGTACCACCGAAA-3’; 19p-TERRA reverse: 5’-GTTCTCCTCAGCACAGAC-3’.

### TERRA half-life assay and qPCR

RBMX-depleted U2OS cells were treated with 5μg/mL actinomycin D (HY-17759, MCE) for 0, 1, 2, 4h. Then the qPCR assay was carried out as described above.

### Chromatin immunoprecipitation (ChIP)

RBMX-depleted or control U2OS cells were cross-linked by 1% formaldehyde and subjected to ChIP assays performed as previously described [61]. Antibodies used were as follows: anti-RNA polymerase II CTD repeat YSPTSPS (phosphor S2) (1:100 dilution, ab5095, Abcam) or anti-IgG (D110502, Sangon Biotech). ^32^P-Labeled telomeric G probe was used to detect the telomere signal in products by slot blot.

### Immunofluorescence *in situ* hybridization (IF-FISH) for detection of R-loop at telomeres

Cells grown on coverslips were swelled in 37°C pre-warmed 0.075 M KCl solution, and incubated at 37°C for 30 min. Then, cells were fixed and washed twice using -20°C pre-cooled methanol: acetic acid (3:1) at room temperature. After that, cells were permeabilized with 0.2% Triton x-100 for 30 min and blocked with 5% goat serum for more than 1 h at room temperature. Fixed cells were incubated sequentially with S9.6 antibody (4°C overnight) and fluorescence-labeled second antibody (room temperature, 1 h).

Soon afterwards, cells were fixed with 4% paraformaldehyde for 30 min at room temperature again, and sequentially dehydrated with 75%, 95%, and 100% ethanol. The coverslips were denatured at 85°C for 5 min, then hybridized with PNA probe (TelC-Alexa488 or TelG-Cy3, Panagene) for more than 2 h at 37°C. After washing steps, the coverslips were stained with DAPI, then visualized and analyzed using Nikon fluorescence microscope.

### DNA-RNA hybrids immunoprecipitation (DRIP) for detection of R-loop at telomeres

DRIP assay was performed as previously reported [62–64] with a slight modification. Cells were trypsinized and collected, then suspended in DRIP lysis buffer (10mM Tris-HCl [pH 7.4], 140 mM NaCl, 5 mM EDTA, 0.5% SDS, PMSF), and subjected to sonication. Genomic DNA was isolated by phenol-chloroform extraction and ethanol precipitation procedure, and treated with/without RNase H (TaKaRa). Nucleic acid samples were suspended in DRIP buffer (50 mM HEPES [pH 7.5], 140 mM NaCl, 5 mM EDTA, 0.05% TritonX-100), and precipitated by incubation with antibody S9.6 overnight at 4°C. The precipitated products were then incubated with Protein A/G Plus-Agarose (Santa Cruz Biotechnology) for 4h at 4°C.

After denaturation, the eluted DNA samples were transferred to a Hybond-N^+^ membrane. The DNA was UV-cross-linked to the membrane before hybridization with G-rich ^32^P-labled telomere-specific probe.

Antibody used: Anti-DNA-RNA-Hybrid Antibody, clone S9.6 (20 μg/mL, MABE1095, Millipore). RNase H pretreated samples were used as a negative control. The pull-down efficiency was calculated as “% of input” after subtracting background signal.

### Transfection of TERRA antisense LNA gapmer

TERRA was masked by an LNA gapmer mediated approach as previously described [41]. The LNA gapmer contains phosphorothioate backbone modifications indicated by “*” in the sequence below.

The position of the LNA modifications and the sequence of the LNA gapmer are designed by Qiagen. At first, U2OS cells were transfected with siNC or siRBMX siRNAs. 24 h post the siRNA transfection, cells were then transfected with LNA gapmer by using Lipo3000 Reagent (Thermo Fisher Scientific) and incubated for 24 h. TERRA or control antisense LNA gapmer includes sequences 5′-T*A*A*C*C*C*T*A*A*C*C*C*T*A*A*C-3′ or 5′-C*A*C*G*T*C*T*A*T*A* C*A*C*C*A*C-3′, respectively.

### Metaphase telomere FISH

Cells were incubated with 2 μg/mL colchicine for 6h to enrich cells at metaphase. Cells were harvested and swelled in 37°C pre-warmed 0.075M KCl solution, and incubated at 37°C for 30min. Then, cells were fixed and washed twice with -20°C pre-cooled methanol: acetic acid (3:1) at room temperature. Cells were transferred to slides. The slides were fixed with 3.7% formaldehyde in PBS for 2min, and treated with RNaseA (10 μg/mL) for 30 min at 37°C After being dehydrated with 75%, 95%, and 100% ethanol, the slides were denatured at 85°C for 5 min, and then hybridized with PNA probe (TelC-Alexa488, Panagene) overnight at 37°C. The slides were washed, stained with DAPI, then visualized and analyzed using Nikon fluorescence microscope.

### Statistics

Statistical analysis was performed using GraphPad Prism version 7. Data are shown as mean ± sd. The unpaired Student’s two-tailed t-test or Two-way ANOVA was used to determine the statistical significance as indicated in the legends (*P< 0.05; **P< 0.01; ***P< 0.001;****P<0.0001).

## Supporting information

S1 Fig RBMX depletion leads to significant increase of TERRA level in HeLa cells. (**A**) Western blot analysis of RBMX knockdown efficiency in HeLa cells. (**B**) TERRA increases in RBMX-depleted HeLa cells. Slot blot was performed to determine the TERRA level, the RNA samples were treated with or without RNase A and RNase T1, with 18S RNA as loading control. (**C**) Quantification of (B). The amount of TERRA was calculated as TERRA intensity/18S RNA intensity, and then normalized to the siNC group. (**D-G**) RT-qPCR analysis of levels of chromosome-specific TERRA transcripts in HeLa cells transfected with siNC, siRBMX-1 or siRBMX-2. Subtelomere-specific primers 6q chromosome (D), 10q chromosome (E), 12p chromosome (F), 19p chromosome (G) were used. GAPDH were used for normalization. Error bars, standard deviations from ≥3 biological replicates. p values, two-tailed Student’s t test (***P <0.001, ****P <0.0001).
(TIF)

S2 Fig Western blot showing the transfection efficiency of indicated siRNA and plasmids. (**A**) Western blot analysis of RBMX and ZCCHC8 knockdown efficiency in U2OS cells. (**B**) Western blot analysis of RBMX and RNase H1 knockdown efficiency in U2OS cells. (**C**) Western blot analysis of ZCCHC8 knockdown efficiency in U2OS cells. (**D**) Western blot analysis of RBMX knockdown efficiency and RNase H1 over-expression efficiency in U2OS cells.
(TIF)

S3 Fig Metaphase telomere FISH detection of fragile telomere signals at the end of chromosomes in the indicated cells. (**A**) Metaphase telomere FISH detection of fragile telomere signals at the end of chromosomes in RBMX-deficient HeLa cells. The fragile telomere signals increase in RBMX-depleted HeLa cells. (**B**) Quantification of (A). The percentage of chromosomes with multiple telomeres signals were calculated. For each group, 500 or more chromosomes were examined. (**C**) Metaphase telomere FISH detection of fragile telomere signals at the end of chromosomes in AZD6738 treated and RBMX depleted U2OS cells. (**D**) Quantification of (C). The percentage of chromosomes with fragile telomere signals were calculated. For each group, 150 or more chromosomes were examined. For panel B, two-tailed Student’s t test was used to determine the statistical significance (*p<0.05, ***p <0.001). For panel D, the Two-way ANOVA was performed.
(TIF)

S1 Table Numerical data underlying graphs in all Figures. (XLSX)
