# Supplementary figures and images for: RBMX involves in telomere stability maintenance by regulating TERRA expression
Source: PLoS Genet. 2023 Sep 27;19(9):e1010937. doi: 10.1371/journal.pgen.1010937 (PMC10529574; doi:10.1371/journal.pgen.1010937)

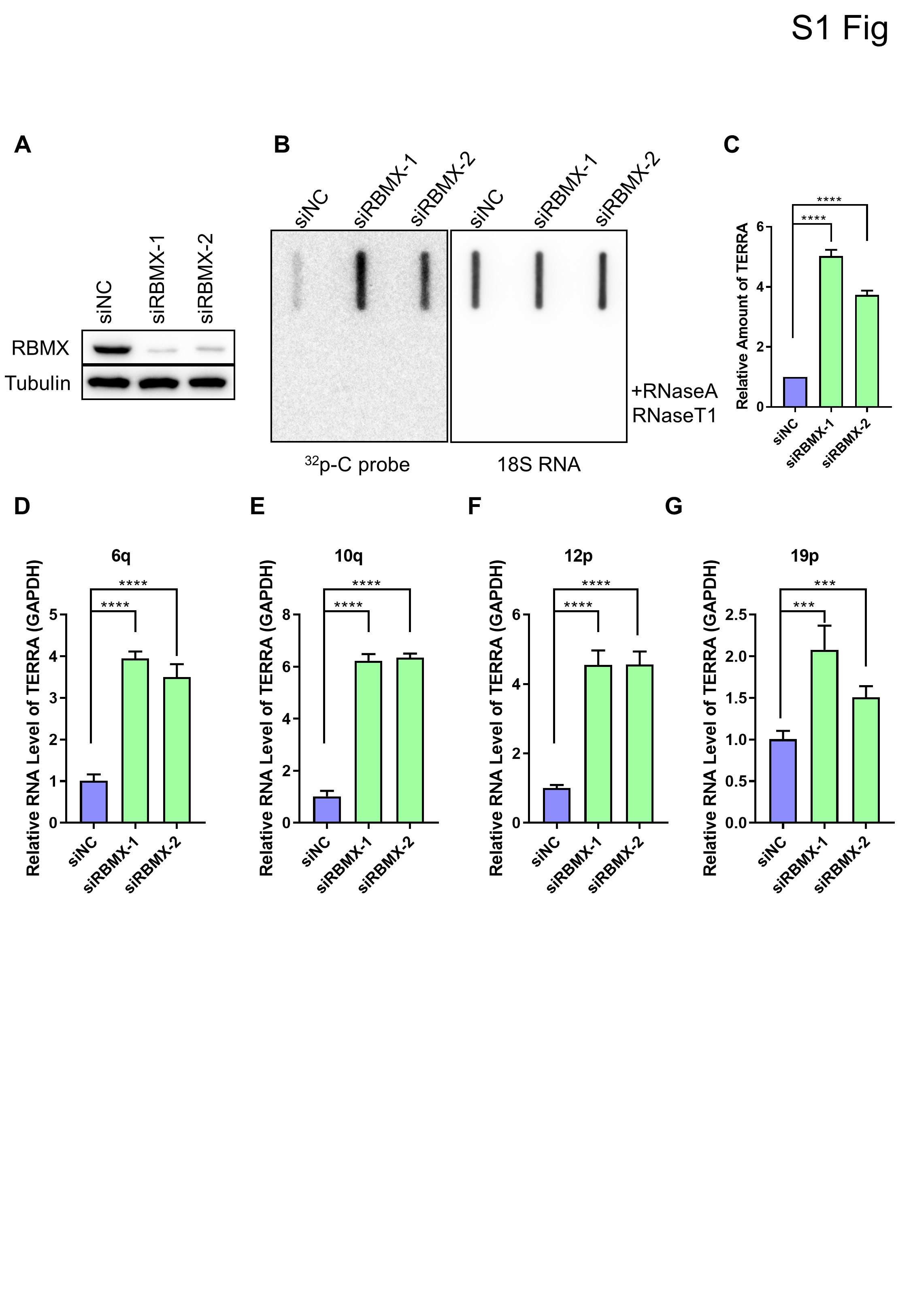

Supplement: S1 Fig — (A) Western blot analysis of RBMX knockdown efficiency in HeLa cells. (B) TERRA increases in RBMX-depleted HeLa cells. Slot blot was performed to determine the TERRA level, the RNA samples were treated with or without RNase A and RNase T1, with 18S RNA as loading control. (C) Quantification of (B). The amount of TERRA was calculated as TERRA intensity/18S RNA intensity, and then normalized to the siNC group. (D-G) RT-qPCR analysis of levels of chromosome-specific TERRA transcripts in HeLa cells transfected with siNC, siRBMX-1 or siRBMX-2. Subtelomere-specific primers 6q chromosome (D), 10q chromosome (E), 12p chromosome (F), 19p chromosome (G) were used. GAPDH were used for normalization. Error bars, standard deviations from ≥3 biological replicates. p values, two-tailed Student’s t test (***P <0.001, ****P <0.0001). (TIF) [file pgen.1010937.s001.TIF]

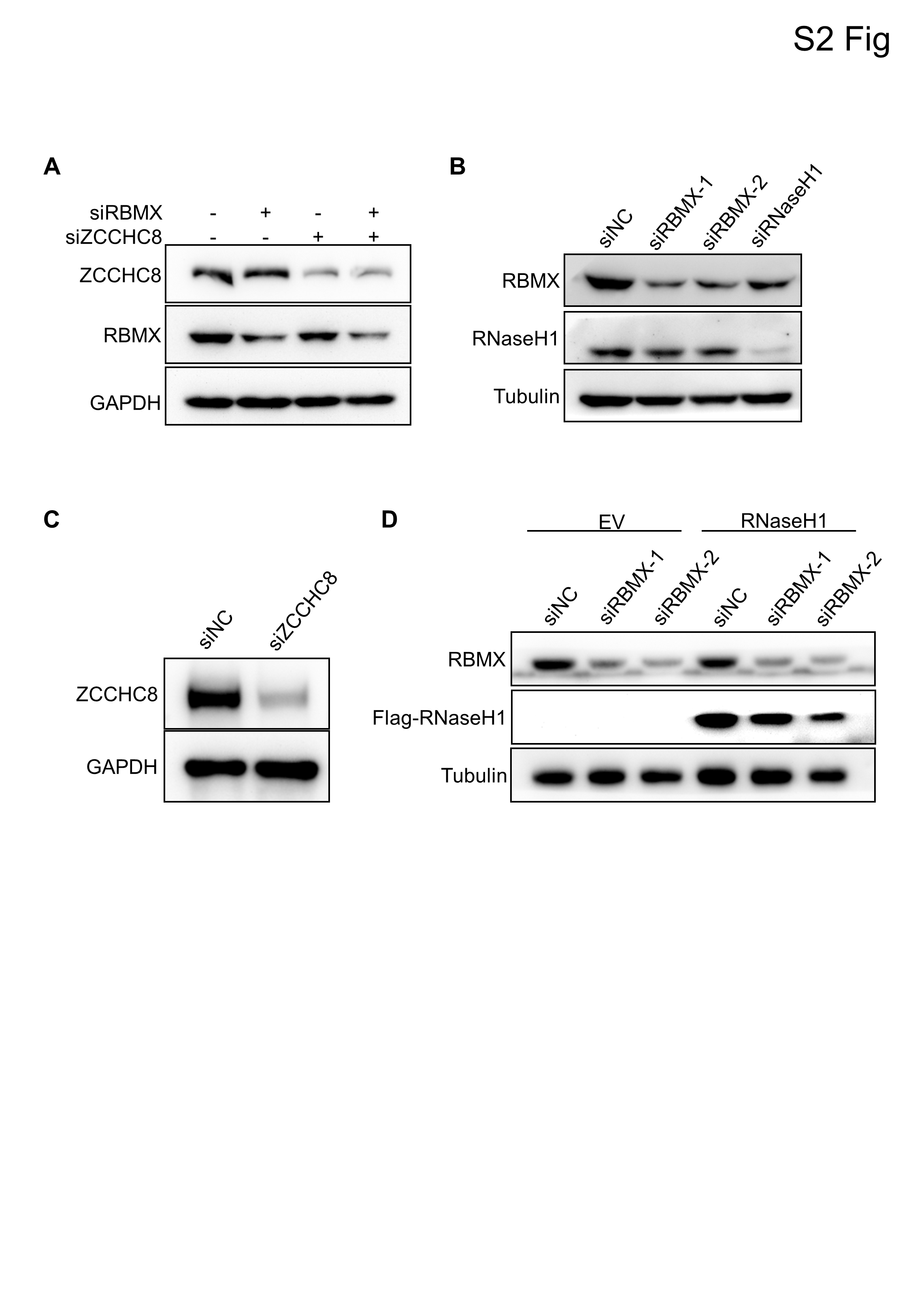

Supplement: S2 Fig — (A) Western blot analysis of RBMX and ZCCHC8 knockdown efficiency in U2OS cells. (B) Western blot analysis of RBMX and RNase H1 knockdown efficiency in U2OS cells. (C) Western blot analysis of ZCCHC8 knockdown efficiency in U2OS cells. (D) Western blot analysis of RBMX knockdown efficiency and RNase H1 over-expression efficiency in U2OS cells. (TIF) [file pgen.1010937.s002.TIF]

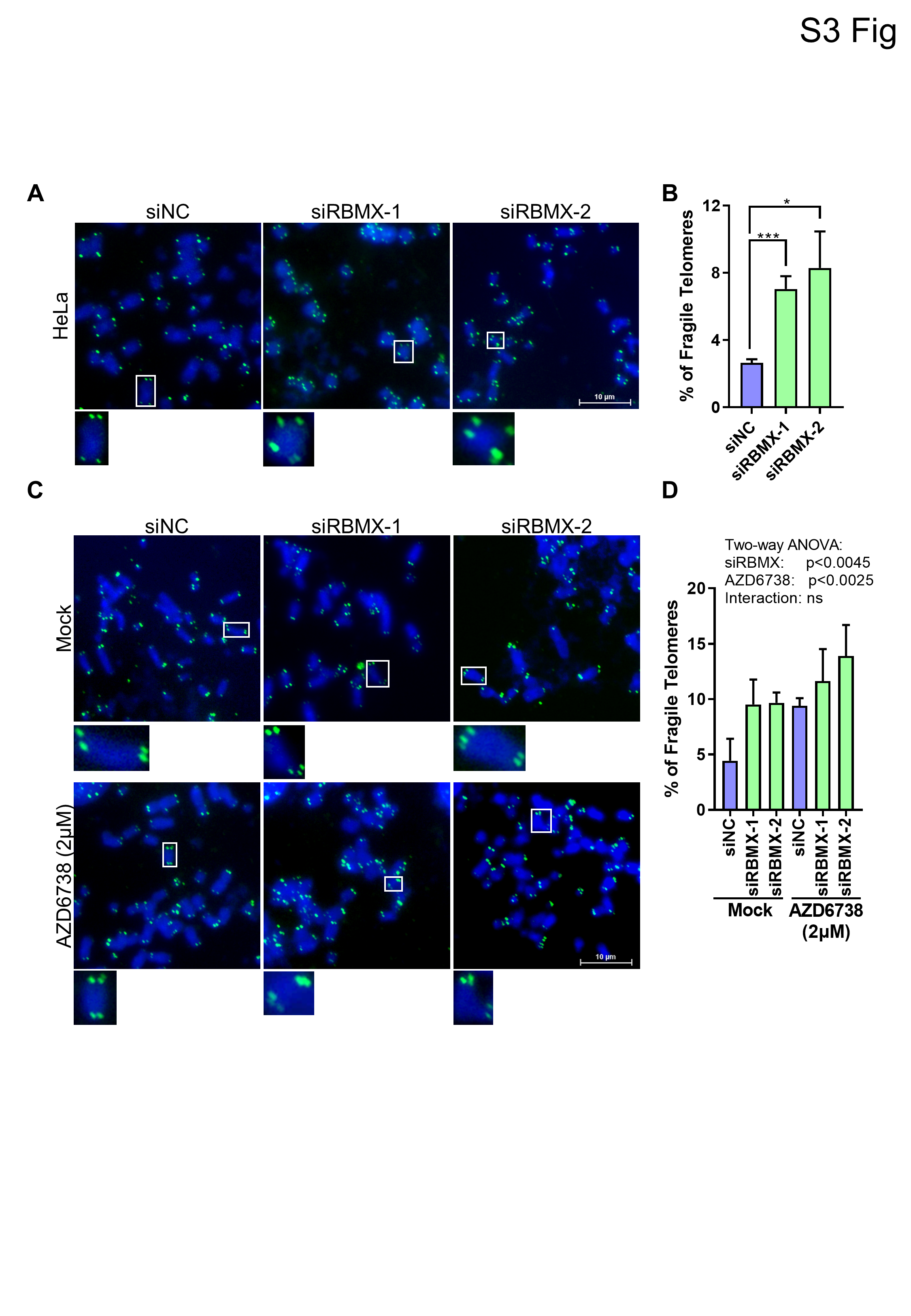

Supplement: S3 Fig — (A) Metaphase telomere FISH detection of fragile telomere signals at the end of chromosomes in RBMX-deficient HeLa cells. The fragile telomere signals increase in RBMX-depleted HeLa cells. (B) Quantification of (A). The percentage of chromosomes with multiple telomeres signals were calculated. For each group, 500 or more chromosomes were examined. (C) Metaphase telomere FISH detection of fragile telomere signals at the end of chromosomes in AZD6738 treated and RBMX depleted U2OS cells. (D) Quantification of (C). The percentage of chromosomes with fragile telomere signals were calculated. For each group, 150 or more chromosomes were examined. For panel B, two-tailed Student’s t test was used to determine the statistical significance (*p<0.05, ***p <0.001). For panel D, the Two-way ANOVA was performed. (TIF) [file pgen.1010937.s003.TIF]
